# The *Xylella fastidiosa* PD1063 Protein Is Secreted in Association with Outer Membrane Vesicles

**DOI:** 10.1371/journal.pone.0113504

**Published:** 2014-11-26

**Authors:** Brittany K. Pierce, Tanja Voegel, Bruce C. Kirkpatrick

**Affiliations:** 1 Department of Plant Pathology, University of California Davis, Davis, California, United States of America; 2 Department of Biology, University of British Columbia, Okanagan, Kelowna, BC, Canada; University of the West of England, United Kingdom

## Abstract

*Xylella fastidiosa* is a gram-negative, xylem-limited plant pathogenic bacterium that causes disease in a variety of economically important agricultural crops including Pierce's disease of grapevines. *Xylella fastidiosa* biofilms formed in the xylem vessels of plants play a key role in early colonization and pathogenicity by providing a protected niche and enhanced cell survival. Here we investigate the role of *Xylella fastidiosa* PD1063, the predicted ortholog of *Xanthomonas oryzae pv. oryzae PXO_03968*, which encodes an outer membrane protein. To assess the function of the *Xylella fastidiosa* ortholog, we created *Xylella fastidiosa* mutants deleted for *PD1063* and then assessed biofilm formation, cell-cell aggregation and cell growth *in vitro*. We also assessed disease severity and pathogen titers in grapevines mechanically inoculated with the *Xylella fastidiosa* PD1063 mutant. We found a significant decrease in cell-cell aggregation among *PD1063* mutants but no differences in cell growth, biofilm formation, disease severity or titers *in planta*. Based on the demonstration that *Xanthomonas oryzae pv. oryzae PXO_03968* encodes an outer membrane protein, secreted in association with outer membrane vesicles, we predicted that PD1063 would also be secreted in a similar manner. Using anti-PD1063 antibodies, we found PD1063 in the supernatant and secreted in association with outer membrane vesicles. PD1063 purified from the supernatant, outer membrane fractions and outer membrane vesicles was 19.2 kD, corresponding to the predicted size of the processed protein. Our findings suggest *Xylella fastidiosa* PD1063 is not essential for development of Pierce's disease in *Vitis vinifera* grapevines although further research is required to determine the function of the PD1063 outer membrane protein in *Xylella fastidiosa*.

## Introduction

*Xylella fastidiosa* (*Xf*) is a gram-negative, xylem-limited plant pathogenic bacterium and the causal agent of a variety of economically important diseases including Pierce's disease (PD) of grapevines, almond leaf scorch and citrus variegated chlorosis [1]–[4]. In nature, *Xf* is transmitted by xylem-feeding insects such as sharpshooters in the leafhopper family Cicadellidae. PD strains exhibit a wide host range although *Xf* does not cause disease on all hosts [4], [5]. Once transmitted to the host plant, *Xf* forms biofilms within the xylem vessels, allowing the pathogen to form a protected niche in which the bacteria can multiply. Bacteria within these protected niches may form large aggregates that effectively plug the xylem element, impede or block transpiration and induce scorching symptoms, similar to what occurs when plants are under water stress. Some plant hosts, such as *Vitis vinifera* grapevines, often die from *Xf* infection [2].

Biofilm formation is a result of density-dependent gene expression, triggered by the process of quorum sensing [6]. Through quorum sensing, bacteria are able to communicate with each other via small signal compounds, which allow the bacteria to recognize population size and mediate the expression of specific genes when bacterial populations reach a threshold concentration [7], [8]. *Xanthomonas oryzae* pv. *oryzae* (*Xoo*), a close phylogenetic relative of *Xf*, is a gram-negative plant pathogenic bacterium and the causal agent of bacterial blight of rice [9]. Although not strictly xylem-limited, *Xoo* colonizes and moves systemically in xylem, similar to *Xf*, and contains many of the same functional genes [10], [11]. Both *Xoo* and *Xf* possess a similar diffusible signal factor (DSF) quorum sensing system. In *Xf*, mutants deficient in DSF exhibit hypervirulence in inoculated grapevines while *Xoo* mutants deficient in DSF exhibit reduced virulence in rice [12], [13]. In both cases, DSF has been shown to play a role in regulation of a variety of virulence factors such as biofilm formation and cell-cell aggregation [14].

Several reports (one of which was retracted) indicate that the *Xoo PXO_03968* –encoded protein and predicted orthologs play a role in quorum sensing, biofilm formation and virulence, [15]–[17]. For example, Qian *et al*. identified a *PXO_03968* ortholog in a proteomic study of the *Xanthomonas oryzae* pv. *oryzicola* (*Xoc*) RS105 secretome. They showed that deletion of the *Xoc ortholog* resulted in reduced biofilm formation and extracellular-polysaccharide production [18]. They also reported that the *Xoc* protein is necessary for full virulence on susceptible hosts. In *Stenotrophomonas maltophilia*, McCarthy *et al*. showed that the *PXO_03968* ortholog (called *smlt0387*) acts as a cell-cell signal to regulate a diverse range of functions, including motility, biofilm formation and virulence [17].

The purpose of this study was to determine the functional role of the *Xf* ortholog of *PXO_03968*, called PD1063. In this study we show that PD1063 is found embedded in the outer membrane and secreted via membrane vesicles. This is supported by recent reports in which the PD1063 homolog, known as *Xoo PXO_03968* was also found embedded in the outer membrane and secreted via membrane vesicles [19]. Our studies indicate that PD1063 plays a role in cell-cell aggregation *in vitro* but does not support a role for PD1063 in regulation of biofilm formation or as pathogenicity factor.

## Materials and Methods

### Bacterial strains and growth conditions

*Xf* Fetzer wild-type strain [20] and the mutant strain *XfΔ1063* (Table 1) were grown on solid PD3 medium [21] without and with kanamycin (5 ug ml^−1^), respectively, for 10 days at 28°C.

**Table 1:** Strains, plasmids and primers.

Strain, Plasmid or Primer	Characteristics and Sequences	Source
**Strains**		
*E. Coli* Top10	F– *mcr*A Δ(*mrr*-*hsd*RMS-*mcr*BC) Φ80*lac*ZΔM15 Δ*lac*X74 *rec*A1 *ara*D139 Δ(*ara leu*) 7697 *gal*U *gal*K*rps*L (StrR) *end*A1 *nup*G	Invitrogen
*Xylella fastidiosa* Fetzer	Wild-type	[20]
*Xylella fastidiosaΔ1063*	*Xylella fastidiosa* Fetzer PD1063::EZ::TN5<Kan-2>Tnp	This study
**Plasmids**		
pCR2.1-TOPO	Kan^R^ Amp^R^, *lacZ*, T7	Invitrogen
pUC18	*bla* (Ap^R^), *lacZ*, *rep*(pMB1)	Thermo Scientific
pCR2.1-PD1063	pCR2.1-TOPO vector with gene PD1063	This Study
pUC18-PD1063	pUC18 vector with gene PD1063	This Study
**Primers**		
PD1063for	TCAGCGCTCTAAAACAATGGCGA	This study
PD1063rev	CACAGCCCGCCTATGGCACA	This study
PD1063chkfor	CAGTTGCAAAACTCCAAGCACAACA	This study
PD1063chkrev	GCGGCGGATCATGAACCTA	This study
Kan2FP-1	ACCTACAACAAAGCTCTCATCAACC	Epicentre
Kan2RP-1	GCAATGTAACATCAGAGATTTTGAG	Epicentre

### Cloning procedures and generation of PD1063-kan

**Figure 1:**
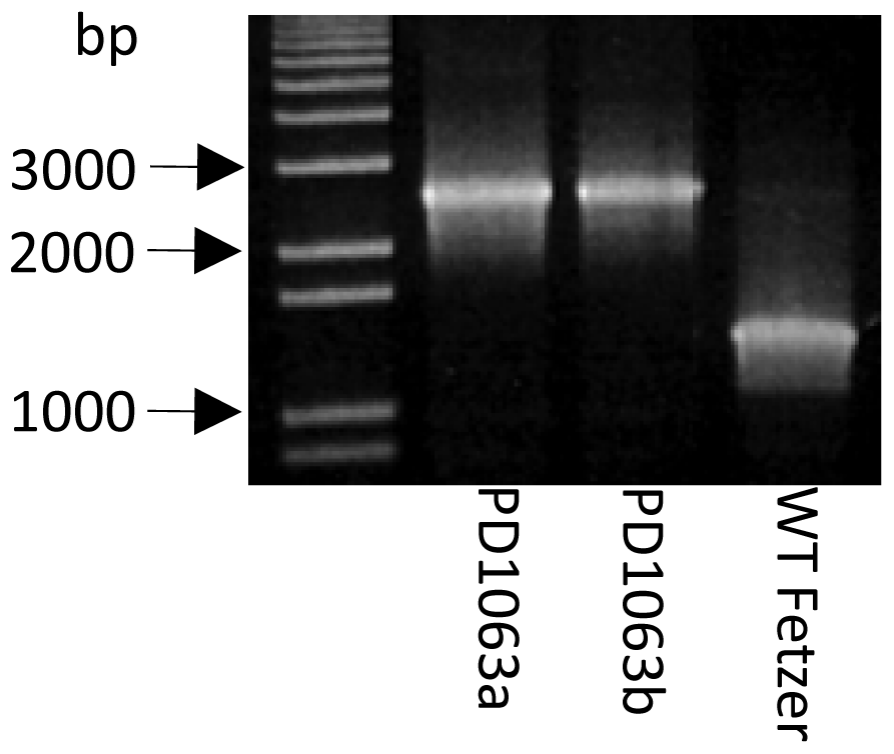
Confirmation of double crossover events in PD1063 mutants. Agarose electrophoresis gel showing confirmation of double crossover in two *Xf* Δ*1063* mutants using primers PD1063chkfor and PD1063chkrev.

PD1063 was PCR amplified from the *Xf* wild type Fetzer genome using primer pairs PD1063for (5′-TCAGCGCTCTAAAACAATGGCGA-3′) and PD1063rev (5′-CACAGCCCGCCTATGGCACA) and the 1015 bp product was cloned into pCR2.1-TOPO vector to generate pCR2.1-PD1063 (Table 1). The plasmid was digested with SacI and XbaI and the resulting fragment was cloned into SacI/XbaI digested pUC18 vector to generate pUC18-PD1063. A kanamycin resistance gene was randomly inserted into pUC18-PD1063 using the EZ-Tn5 <KAN-2> Insertion Kit according to the manufacturer's instructions (Epicentre, California), generating pUC18-PD1063:kan. Sequencing using primers kan-2RP-1/kan-2FP-1 determined the location of the kanamycin cassette insertion was at 102 bp. Plasmid pUC18-PD1063:kan was electroporated into electrocompetent *Xf* wild-type Fetzer cells as previously described [22]–[24], creating the *Xf* mutant *XfΔ1063*. Double crossover mutants were confirmed by PCR analysis of the clones using primer pair PD1063chkfor (5′- CAGTTGCAAAACTCCAAGCACAACA-3′) and PD1063chkrev (5′- GCGGCGGATCATGAACCTA-3′) and results are shown in Figure 1.

### Cell-cell aggregation, surface attachment assays and cell growth

For cell-cell aggregation assays, 10 cultures each of *Xf* wild-type Fetzer and two independent cultures of *XfΔ1063* were incubated in liquid PD3 medium in 15 ml polystyrene tubes in a vertical position without shaking for 10 days. The turbidity (ODs) of the upper culture medium, composed mostly of dispersed cells, was measured using a spectrophotometer at 600 nm. The culture medium was returned to the original tube, the settled aggregate masses were dispersed by pipetting, and the total cell culture was measured (ODt). Relative percentage of aggregated cells was estimated as follows: percent aggregated cells  =  (ODt-ODs)/ODt ×100 [25]. The assay was repeated twice.

For biofilm assays, 10 cultures each of *Xf* wild-type Fetzer and two independent cultures of *XfΔ1063* were incubated in liquid PD3 medium in 15 ml polystyrene tubes in a vertical position without shaking for 10 days. Attachment on the surface walls of the tubes was assessed by a crystal violet staining method [26], [27]. After the incubation period, the PD3 medium was discarded and a 0.1% (wt/vol) aqueous solution of crystal violet was added to each tube, allowed to incubate for 15 min, and rinsed with dH_2_O. The remaining stain was eluted from the bacterial ring using ethanol. The absorbance of the ethanol-crystal violet solution was measured at 540 nm. The assay was repeated twice.

For cell growth assays, 10 cultures each of *Xf* wild-type Fetzer and *XfΔ1063* were incubated in 30 ml liquid PD3 medium in 50 ml polypropylene tubes with shaking at 100 rpm. At the same time every day, for a total of 10 days, the cultures were shaken by hand to disperse clumps and 1 ml of culture was measured using a spectrophotometer at 600 nm. The culture was discarded after being measured. The assay was repeated twice. The data from cell-cell aggregation, biofilm formation and cell growth assays were analyzed non-parametrically using one-way analysis of variance and multiple pair-wise comparison using the Tukey test (SigmaPlot).

### Pathogenicity assay on *Vitis vinifera* Thompson seedless grapevines and *in planta* bacterial population determination

Pathogenicity assays on *Vitis vinifera* Thompson seedless grapevines were performed as previously described [28]–[30]. *Xf* Fetzer and *XfΔ1063* were adjusted to 10^8^ CFU/ml in 1× PBS and two 20 µl drops were inoculated into 15 plants per strain using a pinprick inoculation procedure [31]. PBS served as negative control and 2 replications of the experiment were performed. After the first appearance of symptoms, typically 10 weeks following inoculation, the plants were evaluated for symptom progression biweekly until all plants were dead. A scale from 0 (healthy) to 5 (dead) was used to rate symptoms severity [30]. The data was analyzed non-parametrically using the two-way analysis of variance and multiple pair-wise comparison using the Tukey test (SigmaPlot).

For *in planta* bacterial population determination, petiole tissues from the point of inoculation (POI) and 25 cm above the POI were taken 12 weeks after inoculation and the number of bacteria quantified by culture. Briefly, the petioles were surface sterilized, ground in 2 ml of sterile 1× PBS, and serial dilutions plated on solid PD3 medium. After 7–10 days the number of *Xf* colonies was determined. The data was analyzed non-parametrically using the two-way analysis of variance and multiple pair-wise comparison using the Tukey test (SigmaPlot).

### Generation of anti-PD1063 antibodies

PD1063 antibodies were generated against a 14 amino acid peptide, GDYNKQKFRSIDLKC. The cysteine residue was added to the N-terminus to facilitate keyhole limpet hemocyanin adjuvant conjugation. Peptide synthesis and conjugation was conducted by GenScript USA Inc (New Jersey). The peptide was resuspended in 1× PBS/Freund's incomplete adjuvant (Sigma-Aldrich, Missouri) 50/50 for the first and five booster injections. Immunizations were conducted by the Comparative Pathology Laboratory at UC Davis. One hundred twenty-five micrograms of peptide was injected twice subcutaneously and twice intramuscularly, per injection date, into New Zealand White rabbits on a 10 day schedule. Test bleeds were taken after the fourth and fifth injections and the rabbits were terminally bled three weeks after the fifth injection. Serum was stored at −20°C until used. Indirect ELISA using synthesized non-conjugated peptide was performed to determine the bleed with the highest antibody titer and the optimal working dilution. Activity and specificity of the anti-PD1063 serum were evaluated using pre-immune serum in indirect ELISA and western blots. No non-specific adhesion of polyclonal, pre-immune rabbit antibodies was found (data not shown).

### Isolation of secreted proteins and vesicles

Total secreted protein and vesicles were isolated from *Xf* as described by Voegel *et al*. and Welsch *et al.* [32], [33]. Briefly, wild-type *Xf* Fetzer, *XfΔ1063* and media controls were grown for 5 days in 500 ml PD3 at 28°C. Supernatant was obtained by centrifugation at 3000 g for 10 min and 20 ml of supernatant was placed into dialysis tubing (12-14000 MWCO, Spectrum Chemical Mfg Corp, New Jersey). Samples were dialysed against 1× PBS, 0.1 M EDTA (pH 8.0) and concentrated on polyethyleneglycol bisphenol A epichlorohydrin copolymer 15–20 kDa (Sigma-Aldrich, Missouri) until sample volume was 5 ml. Sucrose was added to the 5 ml concentrated sample to a final concentration of 40% (w/v) followed by the addition of 50 µl 10% SDS, 100 µl β-mercaptoethanol and 2 ml phenol. Samples were vortexed and centrifuged at 800 g for 5 min at room temperature. Approximately 2 ml of the top layer was transferred into a new tube and the centrifugation step was repeated. To collect the protein fraction, samples were divided into two tubes and 10 ml of methanol was added to each tube. Samples were vortexed and centrifuged at 3300 g for 5 min at 4°C. The supernatant was discarded, 10 ml methanol was added to the pellet and the centrifugation step was repeated. The resulting pellet was air-dried and resuspended in 100 µl 1% SDS, 65 mM Tris/HCl (pH 7.0).

For isolation of membrane vesicles, wild-type *Xf* Fetzer, *XfΔ1063* and media controls were grown as previously described and supernatant was obtained by centrifugation at 3000 g for 10 min. Supernatant was filtered through a 0.45 µm sterile filter for elimination of whole cells. Resulting filtrate was centrifuged at 38400 g for 1 h at 4°C to obtain the vesicle pellet and culture supernatant fraction. The pellet was resuspended in 1% SDS, 65 mM Tris/HCl (pH 7.0). Trichloroacetic acid (TCA) to a final concentration of 13% was added to the supernatant fraction. Precipitated proteins were harvested by centrifugation at 15000 g for 15 min, washed with 70% ethanol, air-dried and resuspended in 500 µl 1% SDS, 65 mM Tris/HCl (pH 7.0).

### Isolation of outer-membrane proteins

*Xf* outer-membrane proteins were isolated as described by Voegel et al. and Nikaido [32], [34]. Briefly, one liter volumes of liquid PD3 cultures of *Xf* Fetzer and *XfΔ1063* were centrifuged at 3000 g for 10 min. The resulting *Xf* cells were washed with 10 mM HEPES (pH 7.4) and resuspended in 5 ml of 10 mM HEPES, pH 7.4, containing 0.02 mg DNase ml^−1^, 0.02 mg RNase ml^−1^, 1 mM PMSF and 0.1 M EDTA. Cells were passed three times through a French pressure cell at 900 p.s.i (6.2 MPa) and unbroken cells were removed by centrifugation at 3000 g for 10 min at 4°C. The resulting supernatant was centrifuged at 180000 g for 1 h at 4°C, the pellet was resuspended in 2 ml HEPES buffer and centrifuged at 3000 g for 2 min to remove insoluble material. The supernatant was applied on top of a sucrose step gradient consisting of 2.5 ml sucrose solutions at 55%, 52.5%, 50% and 47.5% (w/w). The samples were placed in a swinging-bucket rotor (Beckman SW-40) and centrifuged at 100000 g for 18 h at 4°C. The resulting bands were collected, diluted with 20 ml HEPES buffer and centrifuged at 180000 g for 1 h. The pellets were resuspended in 100 µl HEPES buffer and stored in 5 µl aliquots at −20°C. Western blot analysis using anti-MopB antibodies [35] (kindly provided by Dr. George Bruening, UC Davis) was used to identify which fraction from the sucrose gradient contained the outer-membrane proteins, MopB protein being the membrane protein reporter.

### Western blot analysis

Proteins were separated by SDS-PAGE on a 12% Tris/HCl polyacrylamide gel in all instances except for identification of outer-membrane fractions with anti-MopB antibodies, which used a 10% Tris/HCl polyacrylamide gel. The proteins were transferred to a PVDF membrane (Whatman, Maine) at 100 V for 1 h in western transfer buffer (25 mM Tris, 192 mM glycine, 10% methanol) and subsequently blocked overnight in 5% nonfat milk in 1× PBS. After washing three times for 15 min with 1× PBS, 0.05% Tween 20, the membrane was probed with a 1∶1600 dilution of anti-PD1063 antisera in 1× PBS for 1 h at 37°C. The membrane was washed three times for 15 min with 1× PBS, 0.05% Tween 20 then incubated with a 1∶1600 dilution of goat anti-rabbit IgG alkaline phosphatase conjugate (Bio-Rad, California) for 1 h at 37°C. The membrane was washed three times for 15 min with 1× PBS, 0.05% Tween 20 and bound conjugate was detected using an alkaline phosphatase conjugate substrate kit (Bio-Rad, California).

## Results and Discussion

**Figure 2:**
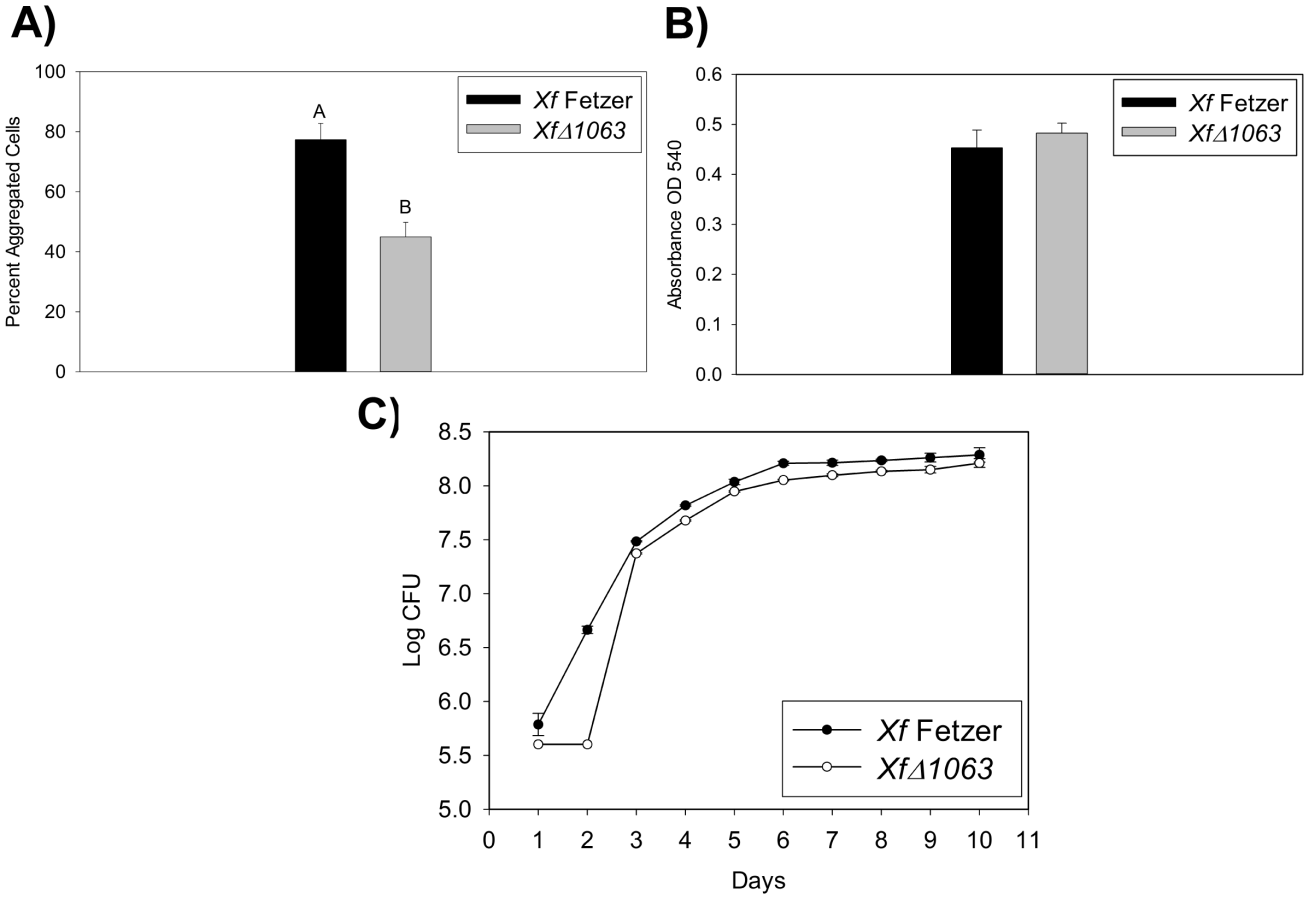
Evaluation of biofilm formation, cell aggregation and cell growth in *Xf*Δ1063 mutants. A) Comparison of cell aggregation by wild-type *Xf* Fetzer, *Xf* Δ*1063* mutant after 10 days growth in static liquid culture. Values shown are mean of 10 samples +/− standard error. The assay was repeated twice and data shown is representative of both assays. B) Comparison of biofilm formation by wild-type *Xf* Fetzer and *Xf* Δ*1063* mutant after 10 days growth in static liquid culture. Values shown are mean of 10 samples +/− standard error. The assay was repeated twice and data shown is representative of both assays. C) Comparison of cell growth by wild-type *Xf* Fetzer and *Xf* Δ*1063* mutant over 10 days growth in liquid culture with shaking at 100 rpm. Values shown are the mean of 10 samples +/− standard error. The assay was repeated twice and data shown is representative of both assays.

*XfΔ1063* mutant cultures formed significantly less (P = 0.003) cell-cell aggregation than wild-type *Xf* (Figure 2A). There were no significant differences between wild-type *Xf* Fetzer and the *XfΔ1063* mutant in surface attachment (biofilm formation) or cell growth (Figure 2B, C). Significance was defined as P<0.05. Cell-cell aggregation is mediated by a variety of factors including hemagluttinin proteins [30], [32]. These factors allow *Xf* to transition from a “sticky”, clumping phase to a movement phase, which is essential for virulence, and often controlled by quorum sensing processes.

**Figure 3:**
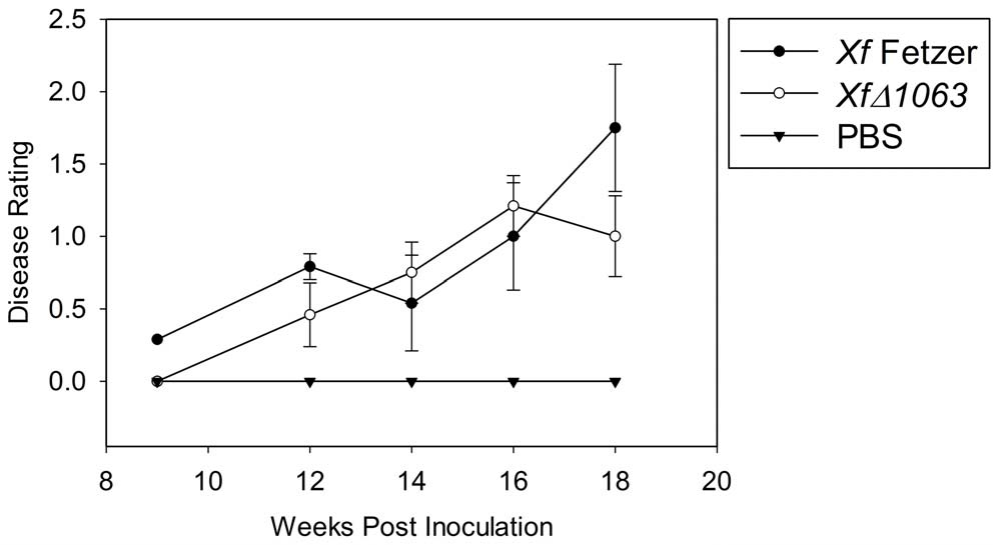
Pathogenicity assay on *Vitis vinifera* grapevines. Disease ratings of greenhouse grown Thompson seedless grapevines pin-prick inoculated with wild-type *Xf* Fetzer, *Xf* Δ*1063* and PBS (negative control) through 18 weeks post-inoculation. Values shown are mean of 15 plants +/− standard error. The assay was repeated twice and data shown is representative of both assays.

We found no significant differences (P>0.05) between wild-type *Xf* and *XfΔ1063* in disease severity after 18 weeks growth under greenhouse conditions (Figure 3). There were no significant differences (P>0.05) in wild-type *Xf* or *XfΔ1063* colony titers isolated 18 weeks post inoculation from the point of inoculation or 25 cm above the point of inoculation (Table 2). These results support recent findings that no statistical differences in populations were observed between the wild type *Xoo* strain PXO99 and the *Xoo PXO_03968* mutant *in planta* [19]. However these studies conflict with a report by McCarthy *et al*. and Qian *et al.* that homologs of this protein have roles in virulence in *S. maltophilia* and *Xoc*, respectively [17], [18]. In the case of *Xoc*, Qian *et al*. also showed a role in cell growth *in planta* [18]. Our results indicate that PD1063 does not play a role in regulation of *Xf* density-dependent processes essential for virulence, except for cell-cell aggregation *in vitro*, in contrast to reports in *S. maltophilia* and *Xoc* [17], [18]. In the case of *S. maltophilia*, the homolog of *Xoo PXO_03968* was required for full virulence whereas with *Xoc*, the homolog was essential for pathogenicity, *in planta* growth and *in vitro* biofilm formation [17], [18]. Cell-cell aggregation was not tested in either of the aforementioned systems.

**Table 2:** Populations (CFU/g of tissue) of *Xylella fastidiosa* isolated from Thompson seedless grapevine petioles 18 weeks post-inoculation.

Strain	POI a	Above POI a
*Xf* Fetzer	1.68 (±1.1)×10^8^	2.03 (±9.3)×10^8^
*Xf*Δ*1063*	1.69 (± 4.5)×10^7^	1.97 (±8.7)×10^8^
PBS	0	0

a Point of inoculation (POI) or 25 centimeters above the POI.

**Figure 4:**
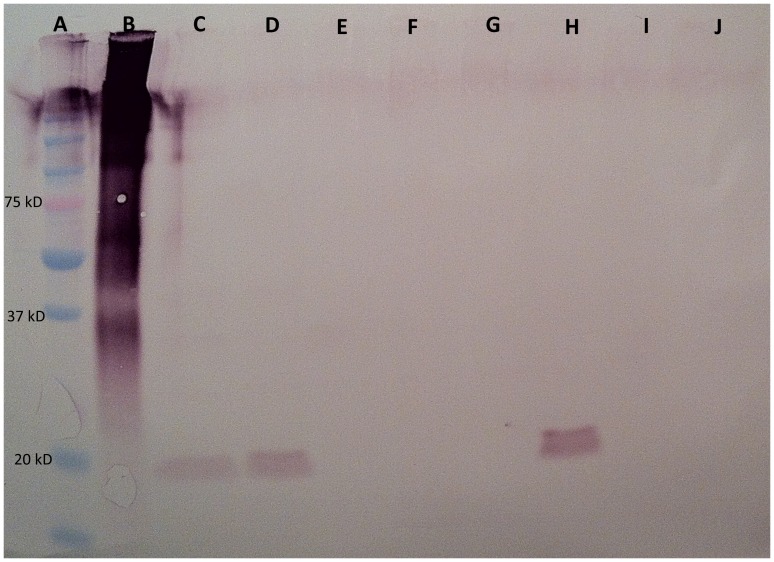
Western blot analysis of *Xf* secreted protein and vesicles. Western blot analysis of isolated secreted proteins and vesicles developed with anti-PD1063 antibodies. Lanes A and B are precision plus protein dual-color standard and positive control (PD1063 Peptide A- keyhole limpet hemocyanin conjugate), respectively. Lanes C and D, *Xf* Fetzer secreted protein; lane E *Xf*Δ*1063* mutant secreted protein; lanes F and G, PD3 media (negative control); lane H, *Xf* Fetzer vesicles; lane I, *Xf*Δ*1063* vesicles; lane J, PD3 media (negative control). Positively stained proteins are found at approximately 19.2 kD in *Xf* Fetzer secreted protein and vesicle samples, the predicted size of processed PD1063 after the signal sequence has been cleaved.

**Figure 5:**
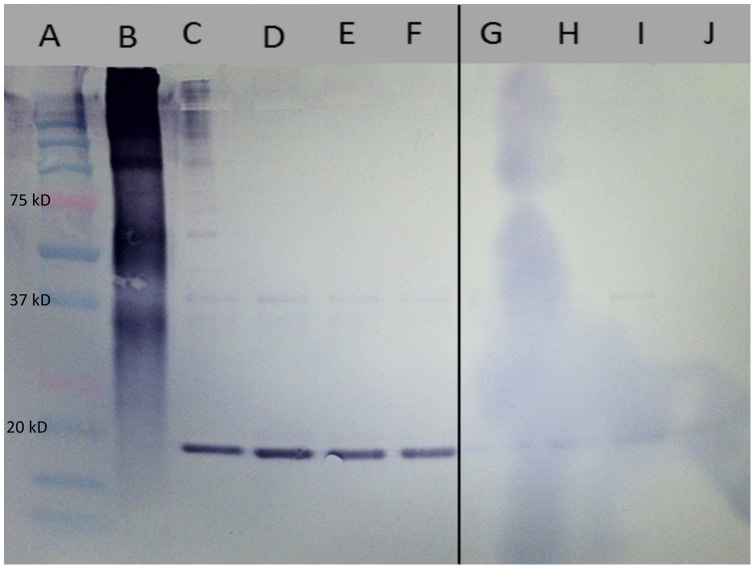
Western blot analysis of *Xf's* outer membrane proteins. Western blot analysis of isolated outer membrane proteins developed with anti-PD1063 antibodies. Lanes A and B are precision plus protein dual-color standard and positive control (PD1063 Peptide A-keyhole limpet hemocyanin conjugate), respectively. Lanes C–F are *Xf* Fetzer outer membrane fractions and lanes G–J are *Xf*Δ*1063* outer membrane fractions. Positively stained proteins are found only in wild type *Xf* Fetzer at approximately 19.2 kD, the predicted size of processed PD1063 after the signal sequence has been cleaved. *Xf*Δ*1063* serves as a negative control since PD1063 is not processed in the mutant strain.

Western blot analysis with anti-PD1063 antibodies on total secreted protein isolated from wild-type *Xf*, *XfΔ1063* and media control identified PD1063 at approximately 19.2 kD only in protein fractions isolated from wild-type *Xf* (Figure 4). Western blot analysis of vesicles isolated from the aforementioned cultures again identified PD1063 only in vesicles isolated from wild-type *Xf* (Figure 4). Outer membrane vesicle production is a general feature of gram-negative bacteria and *Xf* has previously been shown to produce outer membrane vesicles [32], [36], [37]. Western blot analysis on outer membranes isolated from wild-type *Xf* and *XfΔ1063* determined that PD1063 was associated with the membrane only in wild-type *Xf*, again at approximately 19.2 kD (Figure 5). Presence of outer membranes in all fractions was verified with anti-MopB antibodies, MopB being an outer membrane protein marker (Figure S1). Association of PD1063 with vesicles and in the supernatant is predicted by a secretory leader sequence, as identified using SignalP 4.0 [38]. The 19.2 kD protein isolated from the media corresponds to the predicted protein size once the leader sequence has been cleaved [39]. Similar results were also found in the case of *Xoo PXO_03968*, a homolog of PD1063 [19]. Similar to *Xoo PXO_03968*, PD1063 is likely processed through the general secretory system (type II secretion system) [11].

Using protein blast, we found *Xf* PD1063 and *Xoo* PXO_03968 have 48% protein identity. The homologs in *Xoc* and *S. maltophilia* have 48% and 52% protein identity, respectively, to *Xf* PD1063 [40]. It is not uncommon for homologous proteins to have protein identities in this range as there are generally conserved regions of the protein that are important for function. According to PFAM analysis of the *Xf* PD1063 amino acid sequence, the protein is predicted to be an outer membrane protein and is classified as an outer membrane protein β–barrel domain (PF13505) based on the structure of *Escherichia coli* OmpX, PDB entry 1ORM [41], [42]. While we have confirmed that *Xf* PD1063 is located in the membrane, the observed differences in the functional role of the proteins may be due to the differences in amino acid sequence identity. The most important difference observed is the role in pathogenicity, or lack thereof, of *Xf* PD1063 in virulence compared to *S. maltophilia* and *Xoc* [17], [18].

While we found no role for PD1063 in pathogenicity, we did see a reduction in cell-cell aggregation. As predicted, we found PD1063 associated with the outer membrane and outer membrane vesicles. There are a number of factors involved in cell-cell aggregation, including hemagluttinin. With the loss of hemagluttinin, *Xf* was unable to clump and consequentially was hypervirulent *in planta* [30]. The loss of hemagluttinin caused a complete loss of cell-cell aggregation whereas the loss of PD1063 resulted in a small reduction of cell-cell aggregation in *Xf*. We did not see a complete loss of cell-cell aggregation and *Xf* was likely able to clump *in planta* and did not cause an increase in virulence as seen with other non-clumping *Xf* mutants. Since we found PD1063 associated with the outer membrane and vesicles, the protein may be involved in normal vesicle formation in *Xf*. It is possible that the loss of PD1063 has an effect on the membranes of *Xf*, causing it to be less sticky. Other clumping factors, such as hemagluttinin, are still being produced resulting in some level of cell-cell aggregation.

In conclusion, we have investigated the role of the *Xf* PD1063 protein, a homolog of *Xoo PXO_03968*, and found conflicting results with previous reports on homologs of the *Xoo PXO_03968* in *Xoc* and *S. maltophilia*. In *Xf*, we found that the PD1063 protein is not involved in virulence or cell growth *in planta* or biofilm formation and cell growth *in vitro*. This is in contrast to reports of the *Xoo PXO_03968* homolog in other systems. In the case of *Xoc* and *S. matlophilia* reports indicate a role for the *PXO_03968* homolog in biofilm formation and virulence among other density dependent processes. This is not the first time a putative cell-cell signaling protein in *Xf* has exhibited a phenotype different from close relatives. This occurred in the case of DSF where *Xf* exhibited hypervirulence and *Xoo* exhibited reduced virulence when the *rpfF* gene, responsible for DSF production, was mutated [12], [13]. While we were unable to identify a clear role for PD1063, other than in cell-cell aggregation *in vivo*, our research supports recent reports that a homolog of PD1063, *Xoo PXO_03968*, is secreted in association with outer-membrane vesicles. Our results provide further evidence that what seems to be the norm in many other plant pathogenic bacteria is not always the same in the case of *Xf*. Further research is required to understand what role, if any, PD1063 plays in *Xf*.

## Supporting Information

Figure S1 **Verification of** ***Xf*** **outer-membrane protein fraction.** Western blot analysis of isolated outer membrane proteins developed with anti-MopB antibodies. Lane A is precision plus protein dual-color standard. Lanes B-E are *Xf* Fetzer outer membrane fractions and lanes G-J are *Xf*Δ*1063* outer membrane fractions. Positive bands corresponding to the MopB outer membrane protein are seen at 42 kD. This verifies that the outer membrane fraction isolated does in fact contain outer membranes. Lane F is empty.
(TIF)
