# Supplementary figures and images for: The Xylella fastidiosa PD1063 Protein Is Secreted in Association with Outer Membrane Vesicles
Source: PLoS One. 2014 Nov 26;9(11):e113504. doi: 10.1371/journal.pone.0113504 (PMC4245136; doi:10.1371/journal.pone.0113504)

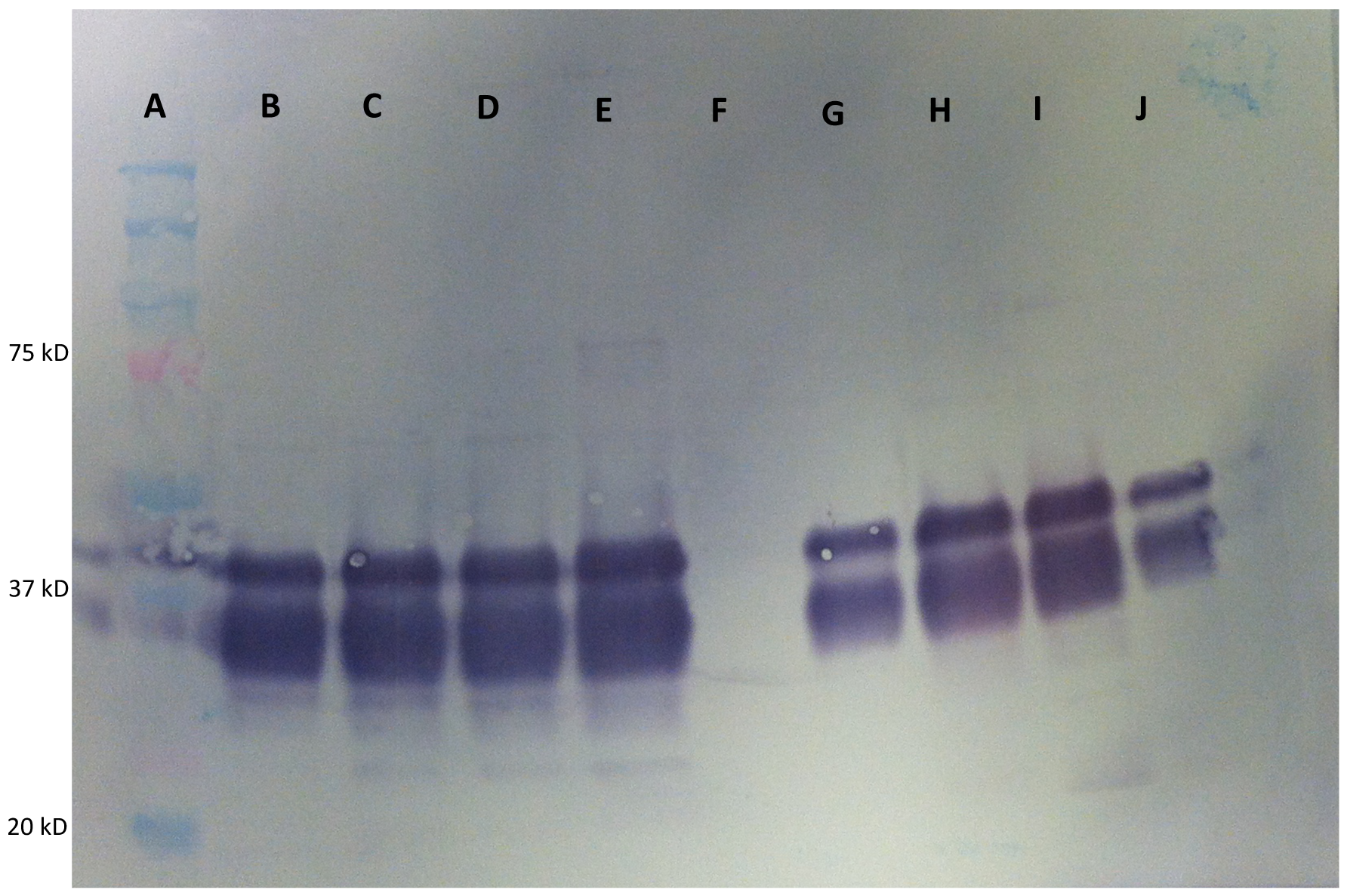

Supplement: Figure S1 — Verification of Xf outer-membrane protein fraction. Western blot analysis of isolated outer membrane proteins developed with anti-MopB antibodies. Lane A is precision plus protein dual-color standard. Lanes B-E are Xf Fetzer outer membrane fractions and lanes G-J are XfΔ1063 outer membrane fractions. Positive bands corresponding to the MopB outer membrane protein are seen at 42 kD. This verifies that the outer membrane fraction isolated does in fact contain outer membranes. Lane F is empty. (TIF) [file pone.0113504.s001.tif]
